# Supplementary material for: Are carbon emissions trading and green financial instruments synergistic? -Comprehensive quantitative research based on content analysis
Source: PLoS One. 2024 Mar 7;19(3):e0298601. doi: 10.1371/journal.pone.0298601 (PMC10919601; doi:10.1371/journal.pone.0298601)
Supplement: S1 Data — (ZIP) [file pone.0298601.s001.zip › Data/地级市Z_F工作报告.zip!/╣π╢1⁄2/│▒╓▌╩╨/└┤╫╘╣1⁄2╓┌║┼í╛┬φ┐╦╩2╛▌═°í┐_2014.docx]

各位代表：

现在，我代表市人民政府向大会作工作报告，请予审议，并

请市政协委员和其他列席人员提出意见。

2013 年工作回顾

2013 年，是我市抢抓机遇加快发展的一年。市政府在省委省

政府和市委的正确领导下，在市人大和市政协的监督支持下，紧

紧依靠全市人民，围绕建设“富裕潮州、美丽潮州、文明潮州、

幸福潮州”的目标任务，深入贯彻党的十八大和习近平总书记视

察广东重要讲话精神，以“三个年”活动为抓手，扩内需谋发展、

优环境强投入、兴实体推转型、惠民生促稳定，实现经济社会平

稳较快发展，基本完成市十四届人大三次会议确定的主要目标任

务。全市完成生产总值 780.3 亿元，比上年增长 11%；人均生产

总值 28837 元，增长 10.5%；地方公共财政预算收入 37.1 亿元，

增长 16.1%；固定资产投资 253.6 亿元，增长 19.3%；社会消费品

2

零售总额 354.1 亿元，增长 11.6%；外贸出口 27.8 亿美元，增长

3.2%；居民消费价格指数增长 2%；人口自然增长率为 6.12‰；城

镇登记失业率控制在 2.35%以内；城镇居民人均可支配收入增长

11.5%；农村居民人均纯收入增长 11.8%。

过去一年，我们着重抓了六方面工作：

一、全力以赴稳增长

面对严峻经济形势，坚持开拓创新、攻坚克难，全力推动经

济加快发展。一是加强扶持稳增长。开展“民营企业服务年”活

动，出台 32 条扶持措施，开通服务直通车，加大信贷资金投放，

支持企业发展。全年安排市级财政扶持资金 3690 万元，帮助企业

申获上级资金 2.45 亿元。金融机构本外币贷款余额 323 亿元，增

长 11.3%，存贷比为 35.1%。二是扩大需求稳增长。57 个市重点

项目完成投资 82.8 亿元，占年度计划 91.2%。建成国际陶瓷交易

中心一期工程，举办潮州国际陶瓷交易会，组织 110 家企业参加

“广货网上行”活动，提升“潮货”影响力，规模以上企业内销

比重达到 80.5%。积极做好欧盟日用陶瓷反倾销应对工作，支持

企业“走出去”，新增境外投资企业 3 家，我市被认定为中国建筑

卫生陶瓷出口基地、出口陶瓷产品质量安全示范区。三是壮大产

业稳增长。积极实施产业倍增计划，大力培育大型骨干企业，新

登记私营企业 1462 户，新增“个转企”536 户，规模以上企业增

至 865 家，年产值超亿元企业达到 294 家，规模以上工业增加值

277.5 亿元、增长 16.5%。支持企业股份制改造，推动企业上市融

资，已上报证监会审批、进入券商辅导的企业各 3 家。完善招商

3

引资项目库，多渠道开展对外招商，实际利用外资 1.01 亿美元。

二、坚定不移促转型

坚持抓创新、调结构、促转型、提质量，着力做大做强实体

经济。一是积极发展现代农业。全力应对强降水和“天兔”等强

台风影响，推进高标准基本农田建设，农业生产保持总体稳定，

超额完成省的粮食考核任务，国家级万亩水稻高产示范点增至 4

个，建成农田整治改造项目 19 个、省级“菜篮子”产品生产基地

16 个、农业部水产健康养殖示范场 7 个、单丛茶现代化产业基地

3200 亩、深水网箱养殖产业园 72.84 公顷、农民专业合作社 560

家。二是改造提升特色产业。大力实施产业转型升级技术改造，

组织申报省级以上科技项目 163 项，完成工业技改投资 26 亿元、

增长 32%。加快科技创新服务平台建设，投用省水族养殖器材和

鞋产品 2 个检验站，新认定省级专业镇 2 个、省工程技术中心 8

个，与 2 家科研院校建立了产学研战略合作关系。大力实施质量

强市战略，新参与各类标准制修订 25 项，我市被确定为“国家

知识产权试点城市”。加快培育战略性新兴产业，省市共建新型环

保包装材料产业基地获得认定，新增国家技术创新示范企业 1 家、

高新技术企业 2 家。三是发展壮大临港产业。以港口为依托，加

大招商引资力度，促进重点产业项目建设增效，华能风电、海山

风能和粤东水产品物流中心等项目建设进展顺利，临港工业实现

增加值 32 亿元、增长 4.1%，潮州港货物吞吐量 1050.6 万吨、增

长 10.5%。四是大力发展旅游产业。完成《潮州国家历史文化名

城保护规划》编制，开展韩文公祠、广济桥和紫莲森林度假村创

4

建 4A 景区工作，筹划建设大凤凰山文化生态旅游产业园，“潮州

老城古民居建筑群”被列为全国重点文物保护单位。加强厦深铁

路城市旅游联盟合作，开展客源市场促销推介，做好“引客入潮”

文章。全市接待海内外游客 590.4 万人次，实现旅游收入 89.9

亿元，分别增长 20.1%和 20.2%。

三、扎扎实实优环境

大力开展“基础设施会战年”活动，突出推进实施 20 个交通、

园区、港口重点项目，进一步优化发展环境。一是加强交通基础

建设。完成厦深铁路潮汕站和饶平站建设配套，按期通车运营。

推进潮惠高速公路、S222 线、S232 线、饶平环城北路等项目，开

工建设韩江东西溪大桥、潮州大桥和韩江大桥维修工程。新改建

县乡公路 52.2 公里、通村公路 215.7 公里，切实改善农村出行条

件。二是加强园区基础建设。基本完成径南工业园一期工程，已

招商项目 31 个、总投资 66 亿元。加快临港产业园、潮安特色产

业基地等园区规划建设，临港产业园被认定为省循环经济工业园，

潮州港临港物流集聚区被列为省现代服务业集聚区，有效提升产

业承载能力。三是加强港口基础建设。逐步完善进港大道、亚太

码头各项配套，抓紧做好小红山码头复建、大唐电厂煤码头、潮

州港公用航道及西澳港区等项目前期工作，潮州港口岸核心能力

建设通过国家验收。四是加强生态保护建设。启动新一轮绿化潮

州大行动，建成绿道网 31.4 公里、森林碳汇工程 7.68 万亩、生

态景观林带 95 公里。巩固提升韩江综合整治成果，实行河砂统采

统销。深入推进“美丽乡村、环卫先行”活动，新增市区垃圾压

5

缩站 5 座，建成农村生活垃圾“一镇一站、一村一点”处理设施。

加大污染减排工作力度，新建镇级生活污水人工湿地 3 个，完成

大唐电厂 1－4 号机组脱硝改造任务，划定高污染燃料禁燃区、高

污染高排放机动车限行区和禽畜养殖区。五是加强水利能源建设。

继续推进沟尾溪、内洋南总干、河内湖等涝区和中小河流综合整

治，完成引韩济枫应急工程，除险加固 5 宗重点小型病险水库。

强化光纤网络、电力设施等建设。推进实施节能降耗重点项目，

单位生产总值能耗完成年度控制目标。

四、统筹城乡抓提升

围绕提升城市辐射能级，加快中心城区扩容提质，推进城乡

区域一体化发展。一是加快潮州新区建设。完成《潮州新区总体

规划》编制，并经省政府批准同意。编制韩东新城起步区概念性

规划，扎实推进韩江东岸综合开发、潮州－中山产业创新中心、

潮州恒大城、市中心医院新院、东方国际茶都等项目，铺开新区

建设。二是加快老城区改造。完成古城区控制性详细规划编制，

批准“三旧”改造方案 1879 宗、面积 5.52 万亩，加快上埔村整

村改造步伐。继续推进市区未开通道路的贯通工作，加快外环路

市区段、枫韩线、护堤路等建设。开展古城区部分街巷改造，启

动“潮州新天地”项目。不断完善城市管理体制，抓好市区广告

清理整治，加强城市净化、美化、绿化、亮化工程建设。三是加

快城镇化进程。完成部分县区行政区划调整，顺利实现潮安撤县

设区，市区面积扩大至 1414 平方公里。推进宜居城乡建设，支持

饶平优化县城功能布局，建成一批名镇、名村和生态示范村。加

6

强土地整治规划编制，农村集体土地确权登记发证工作通过省的

验收。严格实施土地利用规划，顺利完成“五斗山”等征地拆迁

工作，确保城乡重点建设用地需求。四是加快城市群协作。积极

参加第五届粤东侨博会，推进落实汕潮揭同城化项目，实现交通

客运无缝对接、医保联网结算、就业服务一体化。加紧落实中山

对口帮扶潮州各项起步工作，深化与珠三角地区的合作。与厦门

市缔结为友好城市，积极融入海西经济区。

五、尽心尽力惠民生

坚持以人为本，积极发展民生事业。全市财政民生支出 60.3

亿元，占公共财政预算支出的 70.1%，基本完成十项民生实事各

项目标。一是抓好就业社保工作。新增城镇就业 3.2 万人，培训、

转移农村劳动力 1.65 万人和 3.44 万人。努力扩大社会保险覆盖

面，全市参加职工养老、失业、医疗、工伤、生育保险人数分别

增长 7.2%、3.8%、7.4%、4.1%和 7.4%，城乡居民养老保险、医疗

保险参保率分别巩固在 99%和 98%以上。切实增强社会保障能力，

启动实施大病保险，企业退休人员平均养老金提高 10%。二是抓

好解困工程。动工建设市残疾人康复中心大楼，新安置残疾人就

业 529 人。新开工保障性住房 1077 套、基本建成 1490 套，完成

农村低收入住房困难户住房改造 2555 户。推进农村饮水安全工

程，新增受益人口 12.5 万人。全面铺开新一轮扶贫开发工作，努

力提高社会救助水平。三是抓好惠民实事。继续完善公共文化体

育设施，新建成一批公共电子阅览室、数字影院。大力推进全国

文明城市创建工作，广泛开展“文化走亲”等惠民活动，成功举

7

办市第十五届运动会，活跃群众文体生活。市博物馆升级为国家

二级博物馆。深入开展教育创强工作，枫溪区和 4 个镇通过省创

强验收，义务教育标准化学校覆盖率达到 59.7%，公办幼儿园占

比提高到 31%，中考招生实现“三统一”。继续深化医药卫生体制

改革，逐步完善基层医疗卫生服务体系，完成市第三人民医院扩

建工程，建成平价医院 2 家。加强人口计生服务管理，开展免费

孕前优生健康检查。四是抓好其他工作。密切对外关系，与美国

旧金山市等结为友好城市。完善食品药品监管体制，继续推动人

事、统计、法制、侨务、贸促、物价、粮食、打私、人防、气象、

防震、地方志、档案、对台、拥军优抚、民兵预备役、老龄、民

族宗教、妇女儿童等工作取得新成绩。

六、多措并举转作风

以建设人民满意的服务型政府为目标，加强作风建设，加快

职能转变，提升服务水平。一是推进效能提升。认真贯彻中央“八

项规定”，开展“机关作风转变年”活动，切实整治“庸懒散奢”，

查处违风行为 18 宗 22 人。办好政风行风热线，举办网上接访活

动，开通 12345 投诉举报平台，畅通民意诉求渠道。加快行政审

批制度改革，完成市网上办事大厅建设。二是推进管理创新。加

强社会治安防控，提高路面见警率，严厉打击各类违法犯罪活动，

推进平安潮州建设。重视社区矫正工作，强化矛盾纠纷化解调处。

加强安全生产管理，提升应急管理水平。三是推进依法行政。严

格依法履行职责，自觉接受监督。积极配合做好市人大、市政协

组织的评议、调研和视察活动。认真办理市人大代表建议、市政

8

协委员提案 113 件，满意和基本满意率达 99.1%。扩大政务公开，

完善新闻发言人制度。加强公共财政管理，切实减少“三公”经

费支出。强化审计监督和行政监察，加强惩防体系建设，设立公

共资源交易中心，反腐倡廉工作成效明显。

各位代表，过去一年我们经受了严峻考验，取得的成绩来之

不易。这是省委、省政府和市委坚强领导的结果，是全市人民艰

苦奋斗的结果，是市人大、市政协监督支持的结果，也是驻潮部

队、武警官兵、上级驻潮各单位和海内外各界人士鼎力支持的结

果。在此，我代表市人民政府表示衷心感谢并致以崇高敬意！

与此同时，我们也清醒地看到，我市发展中还存在不少问题。

主要是：经济综合实力不强，赶超崛起任务繁重；创新驱动发展

能力不足，投资支撑作用不够明显；资源要素制约亟待破解，节

能减排形势相对严峻；财政减收增支因素增多，保障改善民生压

力较大；社会管理创新有待加强，政府职能转变仍需推进；一些

政府工作人员服务意识不强、办事效率不高等问题依然存在。对

此，我们将采取有效措施加以解决。

2014 年工作安排

今年是贯彻落实党的十八届三中全会精神、全面深化改革的

开局之年，是完成“十二五”规划的攻坚之年，也是加快潮州振

兴发展的关键之年。当前，世界经济总体趋稳，外需状况略有改

善；我国经济长期向好的基本面没有变，深化改革将释放更多的

9

制度红利；省委省政府振兴粤东西北和实施中山－潮州对口帮扶

的决策部署，以及潮州新区启动实施和潮安撤县设区，为我市加

快振兴发展提供了历史机遇和强大动力。但经济发展外部环境仍

存在很多不确定性，以及加快发展与转型升级的协调问题，使我

市发展面临着巨大挑战。对此，我们必须在全面深化改革中坚定

信心、抢抓机遇、善于作为、勇于创新，加快振兴发展步伐。

今年政府工作的总体要求是：全面贯彻落实党的十八大、十

八届三中全会、省委十一届三次全会和市委十三届四次全会精神，

抢抓省振兴粤东西北重大战略机遇，突出追赶全国人均水平“一

个目标”，打造粤东生态环境和社会治安“两个最佳”，依托交通

扩网提速、园区扩能增效、城市扩容提质“三大抓手”，以更加开

放的姿态，着力改革创新，扩大内外需求，推动转型升级，加快

经济发展，保障改善民生，维护社会稳定，努力建设富裕潮州、

美丽潮州、文明潮州、幸福潮州。

综合各方面因素，今年我市经济社会发展主要预期目标是：

生产总值增长 12%、争取增长 14%，地方公共财政预算收入增长

15%，固定资产投资增长 22%，外贸进出口增长 5%，社会消费品零

售总额增长 14%，居民消费价格指数涨幅控制在 3.5%以内，城镇

登记失业率控制在 3%以下，城乡居民人均收入增长与经济增长基

本同步。

围绕上述目标要求，今年重点做好以下九个方面工作：

一、扩大有效需求，促进经济较快发展

把加快发展作为经济工作的突出任务，强化投资、消费、出

10

口的驱动作用，全力推动经济扩量增容。

促进投资有效增长。强化政府投资引导，鼓励支持民间投资，

优化投资结构，扩大产业转型、科技创新、基础设施、产业平台、

社会民生和生态文明等领域的投资。完善重点项目工作协调和推

进机制，抓实 72 个市重点项目，完成年度 111.6 亿元的投资计划。

加强重点项目前期工作，破解项目生成、审批、政策处理等难题，

促进已签约项目落地。建立全市用地指标统筹调控机制，确保重

点项目用地需求。依法推进房屋征收拆迁，为项目落地创造条件。

加强政府投融资平台建设，深化融资手段创新，申报发行企业债

券，解决重点项目建设融资难题。

增强消费拉动作用。依托厦深铁路集聚的人流、物流和资金

流，加快发展高铁经济。创新消费模式，大力发展城市物流配送、

网络购物和连锁经营，争取大型超市投资落户。积极组织适应市

场需求的促销活动，培育城市夜间消费市场，增强旅游、文化、

演艺、餐饮对消费的牵引力。繁荣商贸流通市场，推进农产品冷

藏设施和平价商店建设。扩大商品房销售，促进房地产市场持续

健康发展。加强市场价格监管，规范市场运行秩序。

大力开拓内外市场。落实扩大内需政策，在国内重要城市举

办特色产品展销会，深入推进“潮货全国行”，大力发展电子商务。

支持企业设立营销网点，拓宽产品营销渠道。加快完善国际陶瓷

交易中心，筹划建设潮汕火车站区商贸物流中心、潮安陶瓷产业

流通平台、庵埠食品交易平台、饶平特色产品交易市场、湘桥文

化旅游品市场，创办国际陶瓷采购中心，举办第二届国际陶瓷交

11

易会，服务产业流通发展。推动出口基地和公共技术服务平台建

设，加强出口品牌培育，申报设立公共保税仓，支持企业“走出

去”，增强出口竞争优势，促进外贸出口。扩大先进设备、重要资

源进口，推动外贸平衡。健全外贸预警体系，努力防范外贸风险。

实施产业倍增计划。加大企业扶持力度，进一步优化涉企行

政服务，协调土地、劳动力、财力等资源要素向骨干企业倾斜。

完善信贷担保体系，设立产业发展引导基金，组建村镇银行，为

企业发展提供支撑。抓好市场主体培育，推动中小微企业成长，

新增一批“小上规”企业，扩充“个升企”户数，以量的增大促

进质的飞跃。支持现有企业壮大规模、增强实力，争取有 1－2

家企业上市融资，培育一批具有核心竞争力、经营上规模的骨干

企业，发展 5－6 家年产值超 50 亿元企业。

二、坚持借力发展，扩大对外开放合作

以更加开放的姿态，推动对外交流，密切与周边地区的经济

联系和务实合作。

做实对口帮扶工作。增强机遇意识和主体意识，全面学习借

鉴中山市的先进经验，全方位多领域加强对接合作，促进两市产

业融合发展。争取由中山市主导组建投资开发公司，市场化推进

中山（潮州）产业园建设运营。联合开展园区招商，力争引进一

批大财团、大企业转移落户潮州。加强开发区、专业镇的对口合

作，高起点建设好潮州－中山产业创新中心，配套建设国家（潮

州）陶瓷原材料质检中心，促进优势产业转型发展、做大做强。

举办广东(潮州)工艺美术中山精品展，全面推进教育、人才、

12

旅游等合作。

促进区域合作发展。依托与厦门市的友城关系，加大两地

经贸往来和产业合作，大力拓展闽南市场。深化与漳州的对接

协调，积极推动闽粤经济合作区规划建设，扩大对台经贸文化合

作，加快融入海西经济区。推进汕潮揭同城化，加强交通等基础

设施衔接，落实教育、卫生、文化和广播电视互联互通等惠民项

目，努力建设粤东城市群。

深化对外交流合作。实施互利共赢的开放战略，提升利用外

资水平。发挥侨力侨智和外事资源优势，更好服务经济转型发展。

推进公共外交和人文交流，提高对外开放度。积极参与 21 世纪海

上丝绸之路和中国-东盟自贸区升级版建设。加强城市宣传推介，

塑造潮州良好形象，不断扩大潮州对外影响力和美誉度。

三、着力改革创新，增强科学发展活力

深入贯彻落实党的十八届三中全会精神，大力推进改革创新，

争创发展新优势。

完善市场经济体系。发挥市场在资源配置中的决定性作用，

逐步建设统一开放、竞争有序的市场体系。清理和废除各种行政

壁垒，完成商事登记制度改革，落实“非禁即入”投资政策，探

索开展负面清单管理方式，支持民营企业发展，激发市场主体活

力和创造力。加强社会信用体系和市场监管体系建设，整治规范

市场主体行为。推进第三次经济普查。开展水、垃圾等领域价格

改革。深化投融资体制改革，提升小额贷款公司。完善土地、人

才、产权、技术、信息等要素市场。

13

深化财税体制改革。积极培植税源，优化征管服务，注重公

平税负，增加可用财力。加强预算管理，严格执行预算计划。整

合财力资源，支持振兴发展。增加民生资金支出，推进基本公共

服务均等化。坚持厉行节约，从严控制“三公”经费。

推进社会管理创新。强化基层管理和服务体系建设，增强城

乡社区政务便民、文化体育、医疗卫生等服务功能。完成村、居

换届选举。引导社会组织健康有序发展，扩大政府购买社会服务

范围。推进志愿服务工作。广泛开展群众性文明创建活动，加强

未成年人思想道德建设，做好全国文明城市提名资格测评。完善

民生诉求表达服务机制，规范基层司法所建设，加强社会矛盾纠

纷化解调处，妥善解决信访突出问题。注重源头治理，健全重大

项目“稳评”、“环评”等机制。坚持计划生育基本国策，推动“单

独二孩”政策落实，实施优生惠民工程，促进人口均衡发展。

四、增强特色优势，提升产业发展水平

强化产业支撑，加快转型升级，优化产业结构，增强发展内

生动力，促进产业扩链强体。

建设特色经济示范区。整合政策资源，强化产业导向，调整

产业布局，推动特色产业高端化、集聚化、品牌化发展。坚持创

新驱动，推动工业化与信息化融合，加强知识产权创造和应用，

大力实施一批技术改造项目，提升生产智能化、装备自动化水平。

继续推进质量强市工作，创建陶瓷国家新型工业化产业示范基地，

加强食品软包装等质检平台建设，支持企业参与各类标准制修订、

创建更多名牌名标，增强行业话语权和竞争力。以新型电子材料、

14

环保包装材料产业基地为依托，推进新材料、节能环保、电子机

电、生物制药、药包材等新兴产业发展，带动特色产业改造提升。

引进发展临港大项目。坚持规划引领、设施先行、产业并进，

大力发展临港经济，使临港地区成为活力最强、增长最快的经济

板块。港口码头建设上，推进潮州港小红山码头建设，动工疏浚

潮州港公用航道，扎实做好大唐电厂三期、LNG 接收站、怡华油

品等码头项目建设前期工作。临港产业发展上，大力引进一批重

大临港产业项目，保障大唐电厂、欧华能源等企业正常生产，全

力推进名贵木材集散基地、水产品物流中心等建设项目，力争亚

太物流配送基地、深海网箱养殖设备生产项目、海润冷链物流中

心等开工建设。

加快发展文化旅游业。修编全市旅游发展总体规划，建设提

升牌坊街等一批景区景点，加强重点文物保护和抢修，着力打造

古城文化旅游区，争取新增 4A 景区 3 个，促进古城文化旅游与大

凤凰山生态旅游“双轮驱动”。规划建设美食一条街、美食城，打

造旅游商品特色街区，推进高星级酒店、民居客栈等配套设施建

设，完善旅游“六要素”。依托厦深铁路的带动，加大旅游宣传推

介力度，深化旅游区域合作，鼓励旅行社引客入潮。产业化发展

潮绣、木雕、麦秆画等传统工艺，推动文化与经济融合发展。

扎实推进农业现代化。认真落实中央农村工作会议精神，始

终把“三农”工作牢牢抓住、抓紧抓好。加强中小型灌区配套改

造工程和小型农田水利重点县、水利示范县（镇）项目建设，夯

实农业发展基础。加快农业技术创新和推广，完善农产品质量安

15

全体系，促进农业产业化、基地化、标准化、品牌化发展，全力

稳定粮食生产，提升单丛茶、潮州柑、铁皮石斛、佛手果、深水

网箱养殖等优势特色农业发展水平。创新农业经营管理体制，培

育发展种养专业大户、农民专业合作社，建设大型农产品批发市

场，做大做强农业龙头企业。

五、立足转型发展，推动园区扩能增效

着力提升园区承载能力、产业层次、产出比重和项目集聚度，

更好地发挥引领示范作用。

推动园区规划建设。科学谋划园区发展，做好径南工业园调

整扩容工作，开展远期 3.2 万亩总体规划编制。加快临港产业园、

石化园区、三百门新港区、金狮湾港区等规划修编，抓好各县区

产业园区总体规划、控制性详细规划编制。支持潮安、饶平整合

园区资源，积极申报省级产业园。狠抓园区促建提质，完成潮

州港进港大道通信、照明工程建设，畅通提升园区周边道路，不

断完善园区公共配套设施，营造一流的投资环境。

大力发展园区经济。积极推动各类园区集聚集约发展，支持

高新区完善管理体制，打造区域性创新中心和科技成果转化基地，

壮大电子机电、婚纱晚礼服、生物工程三大产业集群。径南工业

园突出以机械设备、陶瓷新材料等产业为主，推动在建的复兴机

械、万丰工业气体生产等项目尽快竣工投产，促进已签约项目尽

快开工建设。跟踪促进潮安特色产业基地“美新创业园”落地，

合力推进潮安高铁经济区、饶平樟溪低碳工业园等发展，加快形

成功能互补、产业互动的发展格局。

16

加强招商引资工作。牢牢把握发展实体经济这一坚实基础，

在强化支持本土企业的同时，梳理制订招商优惠政策措施，研究

制定产业链招商三年行动计划，积极参加各类招商活动，抓好产

业项目推介，跟踪落实签约项目，力争引进一批经济效益好、产

业辐射力强的重大项目和企业落户。加快建设专业素质强、人员

相对稳定的招商团队，着力提高招商效益。加强土地征收储备和

用地申报，抓好径南工业园二期、市开发区二期 B 区、饶平高堂

火车站区等征地工作，加快潮州港进港公路东片土地开发，为招

商引资创造条件。

六、强化辐射带动，抓好城市扩容提质

以增强城市辐射能级为目标，着力在城乡规划、基础设施、

功能配套和公共服务等方面实现统筹推进、有序发展。

加快韩东新城建设。启动潮州新区市政设施规划编制，完成

韩东新城起步区控制性详细规划，加强韩东新城用地控制和征收。

坚持以重点项目的建设布点，拉开韩东新城起步区发展框架，打

造“三组团”发展格局。突出推进韩江东岸综合开发、潮州恒大

城、市中心医院新院等项目，争取动工建设韩江东岸沿江景观道

路、东方国际茶都，争取启动东厢堤、意东堤防渗并堤路结合工

程和东岸截污排水工程，规划建设潮州东大道，筹建市传媒广电

中心，推动新区全面建设，促进产城融合发展。

优化城区功能布局。抓好各类专项规划编制，实现中心城区

控规全覆盖。完成外环路市区段、枫韩线改造工程，加快北站二

路、宾园路、城新西路、彩虹东路 4 条道路的贯通工作，努力实

17

施绿榕南路、绿榕西路贯通工程。推进公共交通设施、天然气一

张网、污水处理配套管网等项目，保障城市供电、供水、供气安

全。加强“智慧潮州”建设。积极实施“潮州新天地”项目，筹

划修建滨江栈道，推进上埔村、南春路片区等“三旧”改造，完

成古城区部分街巷改造，进一步改变旧城区面貌。完善城市管理

长效机制，实施绿化提升工程，推行环卫清扫保洁和城市广告市

场化运营模式，加大节能照明改造力度，提升城市净化、绿化、

美化、亮化水平。持续开展交通秩序整治，缓解城市交通拥堵现

状。

统筹城乡建设发展。加快新型城镇化步伐，着力增强城镇综

合承载能力。启动滨海新城规划建设。大力发展中心镇，促进人

口、产业集聚。加快户籍制度改革，推动农村富余劳动力向城镇

转移落户。以推进农业产业集中发展、农民集中居住、土地集中

经营为重点，加快新农村建设。推动城市基础设施向农村地区延

伸和覆盖，积极建好农村生活垃圾“一县一场”处理设施，推进

三大涝区整治，动工建设归湖涝区整治工程，加快引韩济饶工程

建设相关工作。深入开展农村环境卫生整治行动，加强宜居城镇、

幸福村居和名镇名村示范村创建工作，大力改善城乡人居环境。

推进交通重点项目。围绕提高城市畅通度和对接度，加大投

资力度，大兴交通基础设施建设。开工建设宁莞高速潮州段，做

好大潮高速潮州段和疏港铁路前期工作，推进潮惠高速潮州段建

设。改造提升省道 S233 线潮安登塘至古巷段、S335 线枫溪广场

至揭阳交界路段、S222 线饶平浮山至黄冈段、S221 线饶平上善段

18

和铁汫线、护堤路、饶平火车站疏站北路等。加快潮州大桥、韩

江东西溪大桥建设，完成韩江大桥维修工程，促进城市有机连接。

启动外环大桥等项目前期筹划工作，争取提前动工建设。

七、重视生态保护，推进美丽潮州建设

牢固树立生态文明理念，着力打造粤东生态环境最佳城市。

加大生态创建力度。全面开展新一轮绿化潮州大行动，建设

市县两级领导造林示范点 57 个，大力实施森林碳汇、生态景观林

带、森林进城围城和乡村绿化美化四大工程，构建森林生态安全

体系。广泛开展生态文明宣传教育，提高全民生态文明意识。

加强环境综合治理。继续巩固提升韩江环境综合整治成果，

开展黄冈河、枫江水污染综合整治。加强水土保持和湿地保护，

推进裸露山体整治复绿。完成柘林湾海洋生态修复项目，加强柘

林湾美丽港湾规划建设。抓好重金属污染防治，推进土壤污染治

理。以更大力度实施“蓝天工程”，开展汽车尾气和城市扬尘综合

治理，实行工业废弃物和危废物规范化处置。健全环境监测监控

体系，提高污染防控能力。

强化节能减排攻坚。严格控制新上高能耗、污染型项目，大

力发展节能低碳产业和新能源、可再生能源。实行单位生产总值

能耗和能源消费总量“双控”管理，加强源头控制。推进以陶瓷

窑炉、电机为重点的节能降耗技术改造，推广先进节能和环保技

术产品。落实污染减排目标责任制，推进城镇污水处理设施及排

污管网建设，动工建设 7 座污水处理厂，抓好规模化禽畜养殖场

改造升级，加快“黄标车”淘汰，积极挖掘减排潜力。

19

节约集约利用资源。加强耕地保护，严格实施年度土地利用

计划，坚决打击违法违规用地行为。加强闲置土地清查处置，盘

活低效用地，做大土地储备总量。抓好水资源节约保护，规范河

砂开采行为。促进海域资源集约使用，合理利用矿产资源。

八、注重民生为先，加快发展社会事业

坚持社会政策托底，切实保障和改善民生，维护社会和谐稳

定，推动幸福潮州建设。

完善社会保障体系。落实收入分配制度改革有关政策，力促

居民收入与经济发展同步增长。实施就业优先战略，积极帮扶重

点群体稳定就业。完善基层服务平台建设，促进充分就业和高质

量就业。做好新一轮农村劳动力技能提升培训工作。推进和谐劳

动关系示范区建设，健全欠薪整治机制。抓好社保扩面征缴，推

进城乡居民养老保险一体化。提升全民医保质量，完善大病保险

机制。建立适度普惠的社会福利体系和城乡统筹的社会救助体系。

统筹发展各项事业。加大教育投入，推进教育创强，优化学

校布局，提升办学质量，推动各类教育均衡优质发展。加强人才

引进和培养，推进人才培训基地、大师工作室建设，加大博士后

科研工作站申报建设力度。抓好文化惠民工程，健全各级公共文

化体育服务设施，推进全民健身活动。开展可移动文物普查。进

一步深化医改工作，增强医疗救援和疾病预防控制能力，促进公

共卫生服务均等化，提升全民健康水平。继续做好民族宗教、外

事侨务、广播电视、新闻出版、国防动员、预备役、对台、人防、

气象、防震、统计、物价、档案、地方志、老龄、妇女儿童、残

20

疾人、红十字等工作，促进各项事业全面发展。

提升公共安全能力。深化平安潮州建设，完善立体化社会治

安防控体系，提高路面见警率和管事率，依法防范和惩治违法犯

罪，着力打造粤东社会治安最佳城市。加强互联网管理，净化虚

拟社会环境。抓好食品药品安全动态监测和长效监管，保障人民

群众饮食用药安全。落实“一岗双责”，推进安全生产专项整治，

坚决遏制重特大安全事故。加强应急指挥系统建设，完善应急工

作体系，做好防灾减灾工作。

办好十件民生实事。把人民对美好生活的向往作为工作方向，

继续办好十件惠民实事。一是办好就业实事。新增城镇就业 2.5

万人，失业人员再就业 4000 人，组织劳动力培训 1 万人、转移就

业 1.5 万人，城镇登记失业率控制在 3%以内。二是办好社保实事。

提升城乡居民医疗保险报销比例；基本实现社会保障卡户籍人口

全覆盖，推进政府公共服务“一卡通”建设；今年 7 月起城乡居

民基础养老金人均标准提高到每月 80 元。三是办好教育实事。

提升教育创强成效，争取新增教育强镇 13 个，义务教育标准化学

校覆盖率达到 68.6%，城乡免费义务教育生均公用经费补助标准

小学、初中分别提高到 950 元、1550 元。四是办好文化实事。建

成开放市图书馆新馆，建设镇村文化站（室）、电子阅览室一批，

完善农村广播电视网络。五是办好卫生实事。在县级医院全面推

进公立医院改革，扩大契约式医疗服务试点范围，加强卫生院及

社区服务中心预防接种规范化门诊建设，将城乡居民医保每人年

补助标准提高到 320 元。六是办好安全保障实事。加快整合建设

21

市食品检验检测机构，推进食品安全镇建设；在中心城区新建消

防栓 474 个、更换维护 171 个；全面完成社会治安高清视频监控

系统（二期）建设；争取地质灾害隐患点搬迁和治理的比例达到

10%以上。七是办好交通实事。改善农村出行条件，新建改建县

乡公路 40 公里、建设通村公路 210 公里，新开通或延伸公交线路

2 条，新建各类公交候车亭 10 个。八是办好环境改善实事。完善

绿道网示范段配套设施，建设生态景观林带 55 公里、森林碳汇工

程 7.68 万亩、绿化美化生态文明村 80 个；深入开展城乡环境卫

生整治，净化美化城乡环境。九是办好解困实事。继续推进新一

轮扶贫开发工作；开工建设保障性住房 900 套、基本建成 450 套，

完成农村低收入住房困难户住房改造 2950 户；建成市残疾人康复

中心，新建社区康园中心 2 个；提高困难群体保障水平，将城乡

低保月人均补差水平提高到 333 元和 147 元，农村五保年供养标

准提高到当地上年度农村居民人均纯收入的 60%，残疾人生活津

贴和重残护理补贴标准分别提高到 600 元/年和 1200 元/年。十

是办好政务惠民实事。完善市县两级网上办事大厅，推进行政审

批事项“减、放、转”，制定公布各级保留的审批事项目录并实行

动态管理，探索审批标准化、推进并联审批，建立“办事工作日”

和“告知承诺”机制，力促政务“一站式”办理，方便群众办事。

九、转变政府职能，加强政府自身建设

顺应振兴发展新要求、人民群众新期待，进一步转变作风、

优化服务、强化监督、提升质效，建设人民满意的服务型政府。

切实转变工作作风。落实中央“八项规定”，开展党的群众路

22

线教育实践活动，加大力度整治“庸懒散奢”，坚决遏制形式主义、

官僚主义、享乐主义和奢靡之风，完善直接联系和服务群众制度，

切实改进政风、文风、会风、作风。深入开展创先争优活动，坚持跟踪问效、督查问责，加强绩效管理、效能监察，营造“敢于

担当、善于落实”的氛围。

积极创新服务方式。深化行政体制改革，切实转变政府职能。

认真履行为民服务职责，真正把功夫用在注重实效、狠抓落实上，

用在突出重点、突破难点上，用在推进发展、造福百姓上。设立

专门解决企业困难的协调机构，对招商项目实行“代办制”、“保

姆式”贴身服务，实实在在为企业排忧解难，推动潮州由“政策

洼地”向“环境高地”转变，增创营商环境新优势。

坚持依法民主行政。运用法治思维和法治方式，深化改革、

推动发展、化解矛盾、维护稳定。完善行政决策程序，健全科学

依法民主决策机制。完善政府信息公开，提高政府工作透明度。

规范行政执法自由裁量权，依法做好法律援助工作。自觉接受市

人大和市政协的监督，广泛听取各方意见，认真办理人大代表、

政协委员建议提案，切实改进政府工作。

不断深化廉政建设。坚持清廉从政、从严治政，按照干部清

正、政府清廉、政治清明的要求，认真落实党风廉政建设责任制，

完善惩治和预防腐败体系，用制度管权管事管人。完善和强化审

计监督制度，加强行政监察。狠抓纠风治乱工作，严厉惩治违法

违纪行为。执行国务院“约法三章”要求，坚持艰苦奋斗，做到

勤俭节约，坚决反对铺张浪费。

23

各位代表，潮州振兴发展的大好时机已经到来，全市上下肩

负着加快发展的历史重任，我们等不得、慢不起。让我们在省委

省政府和市委的正确领导下，改革创新，锐意进取，抓住机遇，

真抓实干，以数据说明成效，以结果评论英雄，让工作成就体现

人生价值，让辉煌业绩造福全市人民！
